# Modal engineering of Surface Plasmons in apertured Au Nanoprisms

**DOI:** 10.1038/srep16635

**Published:** 2015-11-13

**Authors:** Aurélien Cuche, Sviatlana Viarbitskaya, Jadab Sharma, Arnaud Arbouet, Christian Girard, Erik Dujardin

**Affiliations:** 1 CEMES, CNRS (UPR 8011) and University of Toulouse, 29 rue Jeanne Marvig, BP 94347, 31055 Toulouse, France; 2 ICB, University of Bourgogne and CNRS (UMR 6303), 9 av. Alain Savary, BP 47870, 21078 Dijon, France

## Abstract

Crystalline gold nanoprisms of sub-micrometric size sustain high order plasmon modes in the visible and near infrared range that open a new realm for plasmon modal design, integrated coplanar devices and logic gates. In this article, we explore the tailoring of the surface plasmon local density of states (SP-LDOS) by embedding a single defect, namely a small hole, carved in the platelet by focused ion beam (FIB). The change in the SP-LDOS of the hybrid structure is monitored by two-photon luminescence (TPL) microscopy. The dependency of the two-dimensional optical field intensity maps on the linear polarization of the tightly focused femtosecond laser beam reveals the conditions for which the hole defect significantly affects the initial modes. A detailed numerical analysis of the spectral characteristics of the SP-LDOS based on the Green dyadic method clearly indicates that the hole size and location can be exploited to tune or remove selected SP modes.

The control of light transfer through subwavelength volumes still represents a major challenge in the downscaling of optical and electro-optical components for which nanoscale plasmonics concepts offer a most promising gateway^1^,^2^,^3^,^4^,^5^. In this context, extremely small plasmonic architectures obtained by combining self-assembly and surface-directed growth techniques have demonstrated the possibility to reach an impressive spatial control of field confinement and enhancement as well as reduced dissipation^6^,^7^,^8^,^9^. While most studies on metal colloids are conducted on small structures that behave essentially as dipolar optical entities, the synthesis of micrometer-large single crystals that sustain higher order modes provides a means to explore richer SP modal behavior and to envision their tailoring. In particular, the large and ultrathin crystalline platelets considered in this work support high order plasmon modes with resonances in the visible and near-infrared that feature nodes and antinodes localized along the edges of the cavity^10^,^11^. The modal behavior of large platelets is better described by the SP local density of states (SP-LDOS), which is solely governed by the material properties and the boundary conditions resulting from the colloidal shape and is independent of the illumination parameters. In a previous work, we have experimentally monitored the evolution of the SP-LDOS spatial distribution in individual colloidal nanoprisms with size and morphology variations^12^,^13^ or by near-field coupling between two adjacent particles^9^. Henceforth, modal engineering can be envisioned if one can perturb the spectral or spatial distributions of the free electron density oscillations inside the plasmonic cavity. In the conceptual limit where the size of one of the two coupled colloids is reduced down to a Rayleigh particle, the presence of this small polarizable metallic object distorts the SP-LDOS in the remaining plasmonic system^14^.

In this report, we investigate a perturbative approach to rationally design the SP-LDOS that consists in the insertion of a small defect on the edge or inside the colloidal plasmonic resonator by drilling a single hole by focused ion beam (FIB). We show that the spectral and spatial overlap between both resonances, driven by the size and position of the defect, determines whether the native plasmon modal features of the resonator is marginally or significantly modified. We use two–photon luminescence (TPL) maps to image the spatial distribution of the in-plane SP-LDOS in the modified nanoprisms. Numerical simulations of the TPL maps based on the Green Dyadic Method^9^,^15^ account closely for the experimental results and provide a complementary analysis of the spectral modal distribution. In particular, it reveals the different perturbation regimes and SP-LDOS reconstruction in the hybrid hole-nanoprism coupled system. This nanopatterning approach demonstrates the possibly to spatially and spectrally engineer the SP-LDOS at the nanometer-scale, which can be seen as a scaled-down analogy of defect-modified photonic LDOS in photonic crystals that have shown effective light guiding and localization functionalities^16^. Importantly, our FIB-based proof-of-principle is irreversible, yet Babinet’s principle suggests that it could be generalized to a reversible modulation of the SP-LDOS by replacing the hole milling method with the positioning of a complementary nanoparticle that would similarly affect the SP mode distribution^14^,^17^,^18^,^19^.

These nanoparticles are essentially two dimensional (2D) with a thickness of 20 ± 2 nm but lateral dimensions ranging from 500 to 1000 nm. The nanoprisms exhibit a range of in-plane shapes from equilateral triangles (Fig. 1(a)) to regular hexagons with all intermediate truncations. These highly anisotropic yet symmetrical morphologies result from the single crystallinity of the chemically grown gold colloids. The nanofabrication of gap and bowtie antennas by FIB milling into crystalline microplatelets has led to improved plasmonic properties compared to similar structures produced in evaporated metal, since the crystallinity and minimal roughness in the patterned structures were preserved^20^,^21^. In this work, we mill subwavelength holes as it is now well established that such holes perforated in ultra-thin gold films exhibit a broad plasmonic dipolar resonance, centered around 700 nm, overlapping the spectral range of the high order plasmon modes supported by the gold nanoprisms^22^,^23^,^24^. Our choice of a hole geometry also stems from the rich yet fundamental optical physics associated with the response of a single hole microfabricated in a plasmonic metallic film^25^,^26^. This elegant system has been applied to a wide range of nanooptical applications such as optical trapping^27^, extraordinary optical transmission^28^, near field scanning microscopy^29^ or fluorescence enhancement^30^. In addition, the rotational invariance of a circular aperture makes it a genuine in-plane probe of the anisotropy of the colloidal host in particular under a linearly polarized light illumination. Yet, apart from a numerical study of pierced prismatic system^31^, the hole has been almost exclusively studied in extended evaporated films rather than micrometer-sized single crystalline platelets.

## Results and Discussion

We have recently shown that TPL maps result from the convolution of the squared in-plane SP-LDOS with the Gaussian profile of the excitation laser beam and can therefore be exploited as a direct imaging method of the SP-LDOS planar distribution^9^. Notably, when linearly polarized light is used, the recorded TPL image is the partial in-plane SP-LDOS projected along the polarization direction. Notwithstanding the beam convolution, the total in-plane SP-LDOS map can be conveniently reconstructed by summing the images acquired for two orthogonal polarization directions as detailed in reference^9^.

**Figure 2:**
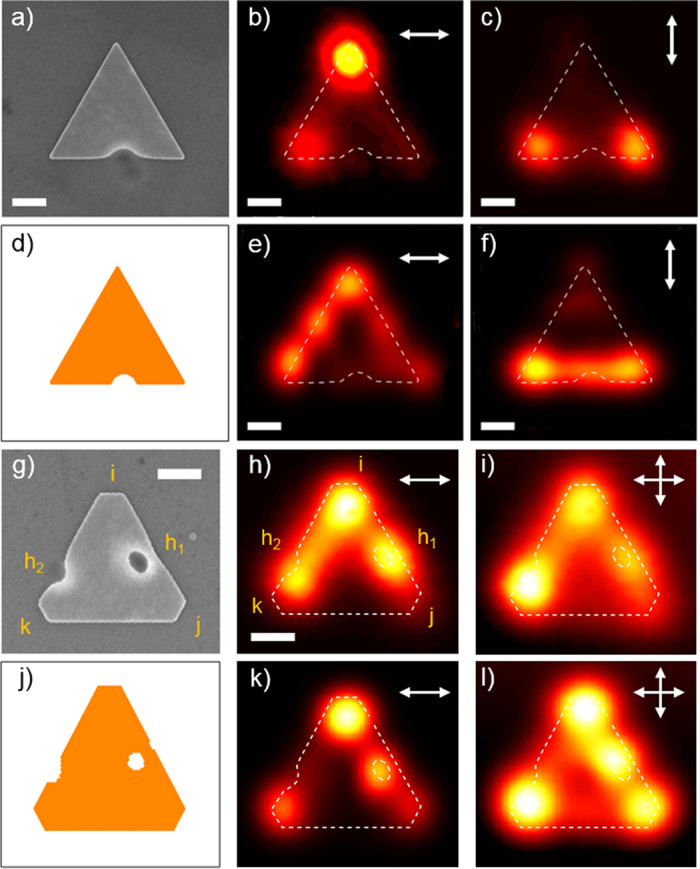
TPL responses of apertured gold prisms. (**a**) SEM image of a gold prism. A single hole has been made across the lower edge of the structure. (**b**,**c**) Experimental TPL map of the pierced platelet. White arrow indicates the linear polarization orientations. (**d**) Geometrical model of (**a**) generated for the simulations. (**e**,**f**) Simulated TPL maps corresponding to (**b**,**c**), respectively. (**b**–**e**) The white dashed line represents the milled prism contour. (**g**) SEM image of a truncated gold prism. Two holes have been milled inside the prism (h_1_) and across its left edge (h_2_). (**h**) Experimental TPL map of the apertured prism. White arrow indicates the polarization orientation. (**i**) Sum of two experimental images recorded for two perpendicular polarizations. (**j**) Geometrical model of (**g**) generated for the simulations. (**k**,**l**) Simulated TPL maps corresponding to (**h**,**i**), respectively. The white dashed line in (**h**,**k**) represents the prism contour. Scale bars are (**a**–**f**) 200 nm and (**g**–**l**) 250 nm.

Figure 2 presents two examples of FIB modified Au nanoprisms. In Fig. 2(a), a sharp triangular prism is perforated with a single semicircular dent on one edge. The TPL response under horizontal (Fig. 2(b)) and vertical (Fig. 2(c)) illumination, essentially shows the characteristics of a pristine triangular platelet, namely the bright spot at the distal apex in the first case and the pair of spots at the proximal apices in the second. Yet, a closer examination reveals that the TPL maps do not match the projection of the perfectly *C*_3*v*_ symmetrical SP-LDOS onto the polarization direction as observed in pristine triangular prisms^12^,^13^. In particular, the intensity of the two spots in Fig. 2(c) is significantly lower than the one in Fig. 2(b). Moreover, Fig. 2(b) presents a secondary spot in the lower left apex but no TPL signal is emitted from the dent position. Identically to a hole drilled in an infinite metallic film^22^,^23^,^24^,^25^,^26^,^32^, we systematically observe a single spot at the hole position which displays no polarization dependence when it is positioned at the center of the prism. The TPL signal emitted by the aperture is systematically more intense than the one of the nanoprism, the spatial distribution of which is only weakly affected by the milling. It therefore appears that shifting the aperture to the edge results in a drastic collapse of its TPL signal below the TPL intensity of the supporting prism (Fig. 2(a–f)) (The normalization procedure is described in the Methods section).

More complex modifications of the SP-LDOS are obtained by combining the effects of several apertures as shown in Fig. 2(g), which presents an example of a truncated triangular Au nanoprism in which two apertures have been placed inside the prism, h_1_, and atop its edge, h_2_. In Fig. 2(h), the TPL map recorded under an excitation polarized horizontally along the (j–k) edge evidences the joint influence of the holes on the response of the gold platelet. It primarily displays a bright hotspot located at the remote apex (i), alike the responses previously reported on pristine Au prisms of similar size^9^. The presence of the aperture h_1_ is revealed by an extra TPL spot at this specific location. Interestingly, its intensity is not much greater than the one on apex (i), as in the case of a central hole, but rather of similar value. Moreover, the edge dent h_2_ triggers luminescence from an area in between apex (k) and h_2_, wherefrom no signal is expected in pristine nanoprisms. We have shown that reconstructed sum images obtained for two orthogonal polarization directions recover the *C*_3*v*_ symmetry of the underlying structure for symmetrical nanoprisms^9^,^12^,^13^. Applying the same approach in Fig. 2(i) to the doubly apertured prism, one clearly observes that the intense TPL spot expected just atop the apices only persists for apex (i) which is the farthest from the FIB fabricated defects. The most intense feature is the one located midway between apex (k) and hole h_2_ while the intensity above hole h_1_ is equivalent to the one in apex (i). Finally, the spot atop apex (j) has almost completely vanished. This recomposed image clearly demonstrates that the presence of the holes can markedly alter the SP-LDOS distribution, either by spatially shifting, attenuating hotspots of the pristine structure or creating new ones. The extent of the modification appears to be related to the spatial (location of the hole with respect to the prism edges) and spectral (its shape and diameter) overlap between the hole and the prism resonances since the alteration induced by the circular hole h_1_ outperforms the one by the split hole h_2_. Importantly, the proximity of the hole with the prism edge affects both the TPL feature of the hole and of the prism as the intensity in h_1_ and (j) are lowered compared to the centered hole or pristine apex. This mutual alteration is not a straightforward superposition but rather results from a spatial and spectral mode coupling as shown in the following.

Deeper insight into the plasmon physics of these complex systems is obtained by applying the GDM numerical tool that we have developed for the realistic simulation of TPL images^9^. The TPL signal is computed from the local electric field **E**(**R**_**0**_, **r**, ***ω***) generated inside the platelet^15^:


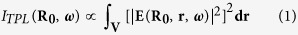



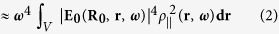


where 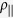 is the in-plane SP-LDOS. Further details on the method are given in references^9^,^15^.

Figure 2(e,k) show the result of the simulated TPL images under horizontal linearly polarized excitation for both geometries and Fig. 2(l) the superimposed images obtained for two perpendicularly polarized illumination conditions for the doubly apertured nanoprism. The agreement with the corresponding experimental images in Fig. 2(b,h,i) is satisfactory. In particular, the effect of the two apertures is fairly accounted for. One should note that the slight experiment-simulation mismatches observed in Fig. 2 originate from the approximate mesh geometry that cannot account for the exact details of the real geometry of the colloids. The location and relative intensities of the main TPL spots are faithfully reproduced and differ significantly from pristine truncated triangular nanoprisms^9^,^12^,^13^. The asymmetry between the responses of apices (j) and (k) is clearly seen in Fig. 2(k,l): the bright spot located above the hole h_1_ appears as intense as the spot in apex (i) with a concomitant reduction of the intensity of the spot in (j). On the contrary, the h_2_ aperture on the edge does not create a separate TPL feature but modifies the location and intensity of the spot near (k). The in-plane spatial distribution of the total squared SP-LDOS of the truncated triangular nanoprism therefore appears as markedly reshaped by the localized FIB milling.

**Figure 3:**
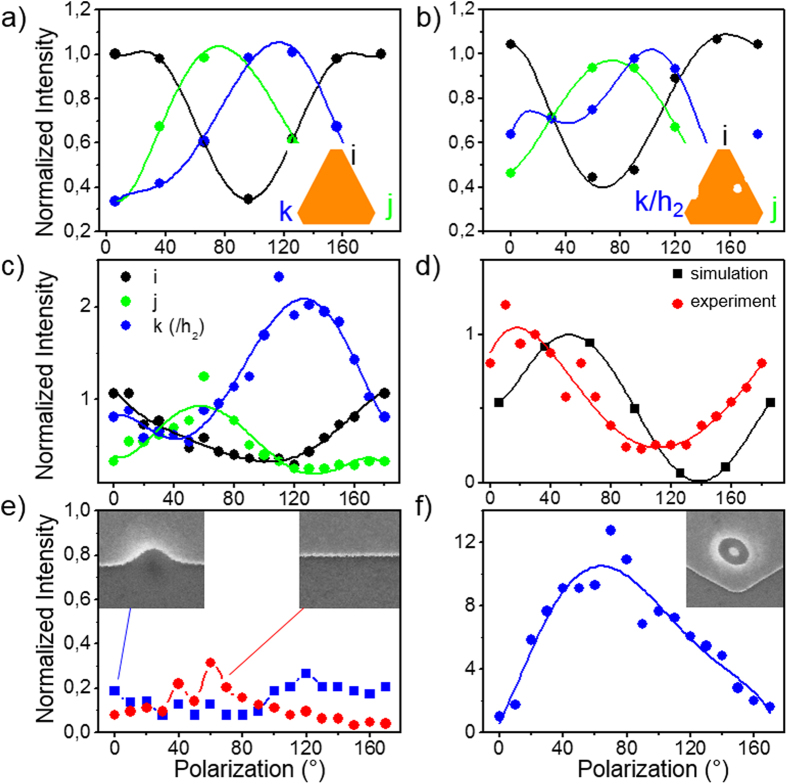
Local variations of the TPL signal with the incident light polarization. The geometries for simulations are shown in insets. (**a**) TPL signal computed at the three apices of an intact truncated prism. (**b**) Same simulations than (**a**) after insertion of two holes. (**c**) Experimental data recorded on the doubly apertured structure. (**d**) Comparison between simulated and experimental TPL signals delivered when the light beam center is located at the h_1_ location. Local variation of the TPL signal recorded (**e**) on a hole milled across the edge and (**f**) on a hole located in the direct vicinity of edges. The local TPL response of an unmodified edge is also presented in (**e**). Corresponding SEM images are in inset (hole diameter d = 100 nm). TPL data in (**e**,**f**) are normalized with respect to the maximum intensity recorded on the prism apices.

In pristine *C*_3*v*_ nanoprisms, a periodical interchange of the maximum TPL intensity over the apices bright spots (i), (j) and (k) is observed when the incident linear polarization direction is swept from 0° to 180° as shown in Fig. 3(a) 9. The distortion of such a typical polarization signature in apertured nanoprisms is illustrated in Fig. 3(b) for the doubly apertured model structure of Fig. 2 and can be compared, in Fig. 3(c), to the dependency of the experimental TPL intensity on the polarization direction. While the global polarization dependency of intensities is preserved after the FIB reshaping, a significant modification of the curve, corresponding to the intensity above the intermediate (k)/h_2_ position (blue data in Fig. 3(b,c)), is observed. For example, the maximum signal is twice higher in (k)/h_2_ than on normalized reference apices (i), (j) and on aperture h_1_, which exhibit approximately the same maximal value. The extinction of the TPL signal when the aperture is perforated on the prism edge is confirmed by the data presented in Fig. 3(e) where only a residual, relatively constant and polarization-independent background is observed. This specific situation could be compared to the insertion of kinks or corners in plasmonic cavities without alteration of the mode signature^33^. On the other hand, the isolated hole h_1_ is responsible for a strong, polarization-dependent signal. Unlike the case of a circular aperture in an infinite film, the intensity in h_1_ can be efficiently turned off by rotating the polarization by 90 degrees (Fig. 3(d)). This distortion of the polarization signature has been systematically observed for holes milled in the direct vicinity of edges as illustrated in Fig. 3(f). Although the residual ellipticity of the aperture could account, in part for this behavior, the polarization dependency of the TPL signal above both apices and apertures suggest a complex hole-prism interaction beyond a mere superposition of individual responses.

**Figure 4:**
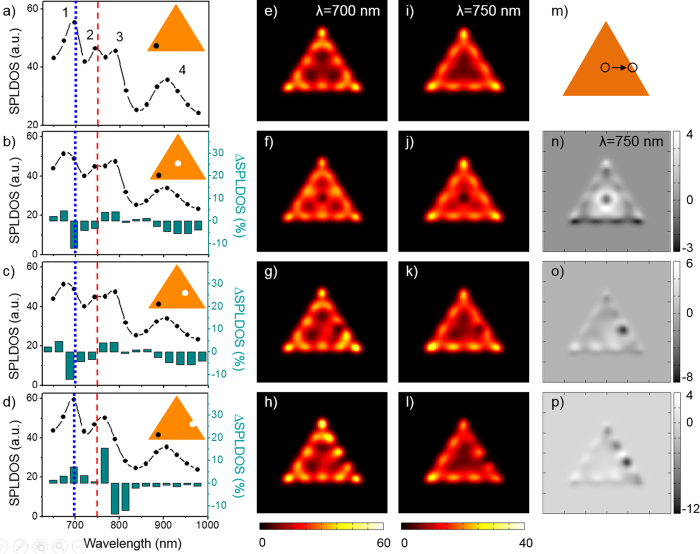
Perturbative vs reshaping alteration of the total in-plane SP-LDOS as a function of defect position. (**a**) Total SP-LDOS spectrum simulated for a 700 nm long reference gold prism without defect. (**b**–**d**) Total SP-LDOS spectrum simulated in the location indicated by the black dot in inset for the same reference gold prism with a defect. Spectral ΔSP-LDOS with respect to reference in (**a**) are plotted as histograms. Geometries and hole positions are displayed in insets. The blue and the red dashed line correspond respectively to the mode 1 and 2 (reference experimental wavelength *λ*_*L*_ at 750 nm). (**e**–**h**) Simulated total in-plane SP-LDOS map at *λ* = 700 nm (mode 1) for the reference 700 nm long gold prism (thickness is 30 nm). (**e**) No hole. (**f**–**h**) With a 90 nm hole inserted in the metallic particle at different locations. (**i**–**l**) Simulated total in-plane SP-LDOS map similar to (**e**–**h**) panels at *λ* = 750 nm (mode 2). (**m**) Actual geometry generated for the simulations and hole positions. (**n**–**p**) Spatial ΔSP-LDOS maps at *λ* = 750 nm corresponding to the difference between maps presented in (**j**–**l**) and reference in (**i**) for each position of the hole.

We further investigated the complex interplay between the plasmonic responses of the hole and the colloidal cavity by extensive SP-LDOS computations in two distinct regimes: the perturbative and the reshaping regimes that both depend on the size and the location of the hole. The scalar function 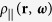 given in Equation 2 not only accounts for the spatial distribution of the total in-plane SP-LDOS, but also reveals the spectral features of the SP-LDOS at an arbitrary location^34^. In order to visualize the spectral and spatial perturbations of the total in-plane SP-LDOS 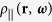 induced by the FIB patterning of a small aperture, Fig. 4 shows the SP-LDOS spectra and maps of a 30-nm thick triangular prism with a side length of 700 nm, perforated with a 90-nm hole in a series of locations between its center and its edge.

In Fig. 4(a), the spectrum, calculated close to the left apex in the intact prism, presents four bands centered at (1) 700 nm, (2) 750 nm, (3) 800 nm and (4) 900 nm of decreasing intensity as the wavelength increases. This multimodal response of the large 2D gold prisms was experimentally observed both in the significant evolution of the TPL intensity maps collected for excitation wavelengths ranging from 700 to 850 nm^9^,^12^ and in EELS maps^10^. In Fig. 4(e,i), the maps corresponding to the excitation of the 700 and 750 nm bands clearly show five and four intensity maxima along the prism edge (respectively 12 and 9 in total along the three edges), which can be attributed to a fifth and a fourth order mode^35^. When a small aperture is embedded in the colloidal platelet, a significant alteration of its spectral and spatial features is observed as shown in Fig. 4(b–d,f–h,j–l). When the aperture is centered, the overall response of the prism is weakened (Fig. 4(b)). More specifically, peak (1) is shifted at higher energy and the composite feature of peaks (2) and (3) is slightly distorted. These variations are more effectively pointed out by considering the relative variation of the spectrum with respect to the one of the pristine prism, ΔSP-LDOS, that is plotted as the inset histogram in Fig. 4(b). The dominant modification at 700 nm is clearly visible in this histogram. While the symmetry of the SP-LDOS map at 700 nm excitation is preserved (Fig. 4(f)), in particular the five maxima remain equivalent on all sides, the modal density is increased around the central region of the prism. The same observation can be done for the mode 2 at 750 nm. For this wavelength, the spatial redistribution of the SP-LDOS is highlighted in differential intensity maps that are obtained by subtracting the reference map (Fig. 4(i)) to the one obtained for the aperture prism (Fig. 4(j)). In Fig. 4(n), one clearly observes an enhanced SP-LDOS around the perimeter of the centered hole that corresponds to its dipolar resonance. This circular signature is spatially disconnected from the edge maxima. In this particular configuration, we therefore observe the superposition of a dominant SP-LDOS of the pristine prism, which is principally located along the edges^9^,^10^, with a small contribution of a circular aperture, which marginally modifies the spectrum around 750 nm and increase the SP-LDOS in an isotropic way, as one would expect from the localized resonance of a hole in a metallic film^26^,^36^. A slight displacement of the aperture position results in a significantly different hole-prism interaction. In Fig. 4(c,g,k,o) the off-centered aperture is characterized by the same spectrum as the centered hole (Fig. 4(b)), yet the increased SP-LDOS on the aperture perimeter has vanished (at 750 nm, see Fig. 4(o)). Notably, the *C*_3*v*_ symmetry of the edge patterns is modified with the reinforcement of the distal apex region at the expense of the regions near the apex on either side of the hole. When the hole reaches the edge of the prism, the entire system is severely perturbed both spectrally and spatially (Fig. 4(d,h,l,p)). The strongest spectral modifications are the reinforcement and low energy shift of peak 1 back to 700 nm and the merging of peaks 2 and 3 into a single band close to the initial position of mode 2 at 750 nm. In the maps of Fig. 4(h,l), an intense feature appears on the side of the aperture position. Moreover, the intensity of the distal apex and sides matches or even exceeds the ones observed on the pristine prism (minimal bright contrast in differential map of Fig. 4(p)), as the proximal edge is significantly attenuated (dark contrast in Fig. 4(p)) Both observations are in good agreement with the two TPL regimes illustrated in Fig. 2. Interestingly, in this resonant configuration, the perturbation of the prism SP-LDOS by the hole milling can be tuned from minimal to drastic depending on the degree of spatial overlap between the localized dipolar response of the hole and an antinode of the high order mode of the cavity. It can reach as much as 80% of the initial density for a small 90-nm diameter hole. In the latter case, the long-range sensitivity of the SP-LDOS is striking as the strong hole-prism coupling at the edge is effectively affecting the SP-LDOS at the most remote point, at distances larger than several plasmonic effective wavelengths.

**Figure 5:**
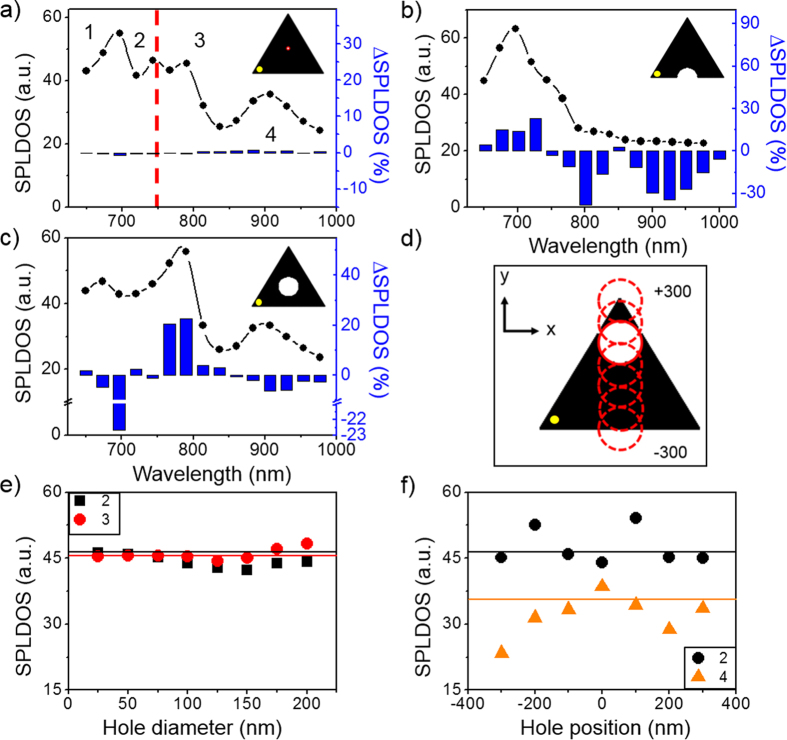
Plasmonic modal engineering in crystalline Au platelets. (**a**) Simulated total in-plane SP-LDOS spectrum for a 700 nm long gold prism with a centered 25 nm diameter hole. (**b**,**c**) Same than (**a**) for different hole positions and a hole diameter d = 200 nm. Geometries and hole positions are displayed in insets. Spectra are calculated at the remote position indicated by a yellow dot. The experimental wavelength *λ*_*L*_ is given as a reference by the red dashed line. Spectral ΔSP-LDOS with respect to the reference 700 nm long gold prism are plotted as blue histograms. (**d**) Scheme of the geometry and successive hole positions (d = 200 nm). (**e**) SP density evolution as a function of the hole diameter for modes (2) and (3). The hole is located at the center of the prism as illustrated in (**a**). Straight lines correspond to mode density for the reference pristine system. (**f**) Total in-plane SP-LDOS evolution as a function of the hole location for modes (2) (black dots) and (4) (orange triangles). Diameter of the hole is d = 200 nm. The positions of the hole are presented in (**d**). Straight lines correspond to mode density for the reference unpierced system.

The transition between the perturbative and the SP-LDOS reshaping regimes also depends on the relative area of the hole and prism^31^. For instance, the impact of large centered holes on the cavity modes of prismatic structures has been discussed in reference^31^. In Fig. 5, we explore the effects of gradually increasing the diameter of a centered aperture from 25 to 200 nm. As the hole diameter increases both resonators are spectrally detuned since the hole resonance is red-shifted^22^. Surprisingly, the sole aperture size expansion is not sufficient to switch from the perturbative to the reshaping regime. Indeed, we observe a spectral shift of peak 1 and an intensity attenuation of peak 4 as the feature of peaks 2 and 3 is gradually distorted by the disappearance of peak 2 and growth of peak 3 (Fig. 5(a,c)). This evolution does not show a drastic transition but rather a continuous evolution similar to the one observed by moving the small 90-nm aperture towards the edge. It therefore appears that the modal structure is little affected by centered holes but major SP-LDOS modification can be obtained by placing an aperture with a diameter of the order of half the wavelength of the SP mode on a prism edge. The comparison of the spectra in Fig. 5(a,b,e) shows that the milling of a 200-nm diameter hole in the center of a 700-nm triangular prism strongly attenuates peaks 1 and 2, but when the hole is milled on the edge, peak 4 is completely suppressed and only peak 1 of the pristine structure is preserved.

## Conclusion

In conclusion, we have demonstrated that the plasmonic signature of 2D crystalline Au platelets with micrometer-size could be modified by the simple perforation of a single subwavelength hole. Pristine prisms are multimodal plasmonic resonators with spatial and spectral characteristics governed by their size and planar shape through their SP-LDOS. Conversely, apertured prisms exhibit two distinct regimes that depend on the size and location of the perforation. The first regime consists in a moderate perturbation of the native spatial and spectral plasmonic characteristics, and occurs when the aperture is centered on areas of minimal SP-LDOS of the pristine prism. The second regime is the long-range restructuration of the SP-LDOS within the colloidal cavity, and occurs essentially when the aperture is adjacent to a prism edge or when the hole size is comparable to the plasmon effective half-wavelength. The strongly coupled hole/platelet system should then be considered as a new entity with a specific plasmonic signature that results from the long range modification of the initial modal density. Experimental two-photon luminescence microscopy and extensive numerical simulations by the Green Dyadic Method were used to assess how the single circular apertures affect the SP-LDOS of the prisms. Initially, the engineering of the SP modal distribution in these large colloidal platelets was shown to foster the development of new ways to integrate optical components for information processing^9^. Our approach opens the way to new strategies for on-demand plasmonic modal engineering in low dissipative crystalline planar colloidal systems for optical information processing.

## Methods

### Au nanoprisms synthesis and sample preparation

The gold nanoprisms are prepared by the reduction of HAuCl_4_ by polyvinylpyrrolidone (PVP) in alkaline conditions. The pH of an aqueous solution that contains 0.143 mM NaNO3, 0.143 mM KI, 0.477 mM HAuCl4 and 19.1 mM PVP (total volume 209.6 ml) is increased by addition of 0.2 ml of 1 M NaOH, diluted in water. The solution is left undisturbed for 18 h. The Au nanoprisms are then re-dispersed in deionized water. A drop of the nanoprism suspension is then deposited onto ITO-coated glass coverslips. The PVP coating is removed by oxygen plasma etching (5 min). Circular subwavelength apertures are milled in pre-selected nanoprisms by focused gallium ion beam (Orsay Physics FIB and Zeiss 1540XB SEM).

### TPL microscopy

TPL images before and after FIB modifications were recorded on a home-built optical microscope coupled to a tunable Ti:Sapphire pulsed laser delivering 120 fs, linearly polarized near-infrared pulses (Coherent Chameleon Ultra II). The mapping experiments have been performed by scanning the sample with a piezo XY stage (Mad City Labs Nano-PDQ250) in the 300 nm FWHM focal spot of the laser tuned at 750 nm with a nominal power of 100 *μ*W measured at the back aperture of the high-numerical-aperture oil-immersion microscope objective (Olympus x100, NA 1.35). The spectrally broad TPL signal was then collected pixel by pixel in an epi-collection geometry through a low-pass filter centered at 650 nm (excluding the excitation at 750 nm) and focused on a photomultiplier tube (PMT; Hamamatsu H7422P-40). Generally, images are normalized with respect to the squared incident power to facilitate a quantitative comparison between them. Nevertheless, when the internal TPL features of a single image are examined, the signals are normalized with respect to the ones acquired on top of the apexes of the unmodified prism (reference).

### Numerical simulations

Our numerical tool is based on the 3D Green dyadic method that allows the precise computation of the local electromagnetic field **E**(**R**_**0**_, **r**, ***ω***) inside any arbitrary 3D metal systems. To this end, the sample volume *V* is described by the discretized model shown in Fig. 2(d) that comprises 10 000 elementary cells distributed over a hexagonal lattice. Then, the generalized propagator **K**(**r**, **r**′, ***ω***) is computed^37^. This propagator contains the total electromagnetic response of the tailored gold structure and therefore enables the computation of the local electromagnetic field **E**(**R**_**0**_, **r**, ***ω***), inside and outside the system under any arbitrary laser excitation associated with the electric field **E**_**0**_(**R**_**0**_, **r**, ***ω***):


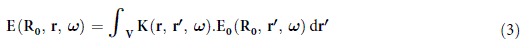


where **R**_**0**_ labels the light beam center and ***ω*** is the angular frequency of the laser.

The TPL signal emitted by the illuminated sample is computed in a second step from this local electric field distribution. The integration of the squared local electric field intensity on the whole structure volume gives rise to the TPL signal expected for a specific position of the Gaussian excitation (see equation (1)). The TPL images are finally computed, pixel by pixel, by scanning the virtual Gaussian light beam on the sample.

## Additional Information

**How to cite this article**: Cuche, A. *et al.* Modal engineering of Surface Plasmons in apertured Au Nanoprisms. *Sci. Rep.* **5**, 16635; doi: 10.1038/srep16635 (2015).

## Figures and Tables

**Figure 1:**
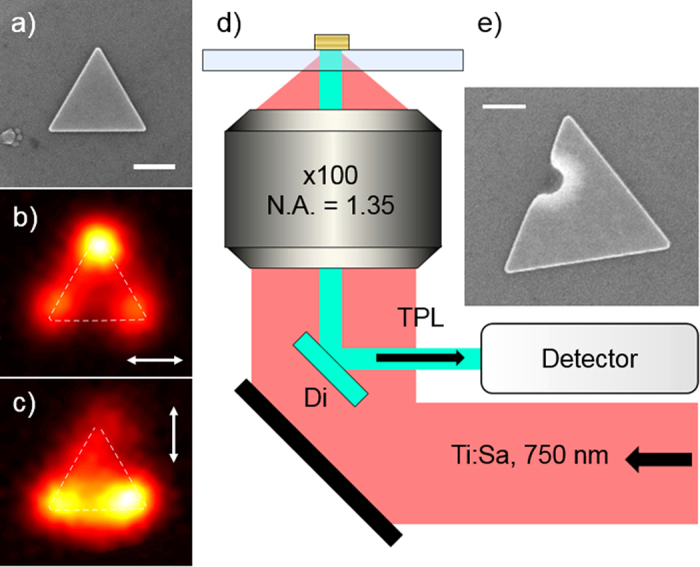
Experimental configuration. (**a**) SEM image, (**b**) TPL map recorded near a pristine gold prism for a horizontal polarization and (**c**) for a vertical polarization. Polarization is indicated by the white arrows. (**d**) Schematic drawing of the home made microscope in which a femtosecond laser beam tuned at 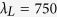 nm excites the photoluminescence. (**e**) Example of a gold prism locally milled by FIB. A narrow gallium ion beam of the FIB is used to indent the side of the prism. Scale bars are 200 nm.
